# Recent Advances in Wayside Railway Wheel Flat Detection Techniques: A Review

**DOI:** 10.3390/s23083916

**Published:** 2023-04-12

**Authors:** Wenjie Fu, Qixin He, Qibo Feng, Jiakun Li, Fajia Zheng, Bin Zhang

**Affiliations:** 1 Key Lab of Luminescence and Optical Information, Ministry of Education, Beijing Jiaotong University, Beijing 100044, China; 2 Dongguan Nannar Technology Co., Ltd., Dongguan 523050, China

**Keywords:** wheel flat detection, wayside signal acquisition method, signal processing method

## Abstract

Wheel flats are amongst the most common local surface defect in railway wheels, which can result in repetitive high wheel–rail contact forces and thus lead to rapid deterioration and possible failure of wheels and rails if not detected at an early stage. The timely and accurate detection of wheel flats is of great significance to ensure the safety of train operation and reduce maintenance costs. In recent years, with the increase of train speed and load capacity, wheel flat detection is facing greater challenges. This paper focuses on the review of wheel flat detection techniques and flat signal processing methods based on wayside deployment in recent years. Commonly used wheel flat detection methods, including sound-based methods, image-based methods, and stress-based methods are introduced and summarized. The advantages and disadvantages of these methods are discussed and concluded. In addition, the flat signal processing methods corresponding to different wheel flat detection techniques are also summarized and discussed. According to the review, we believe that the development direction of the wheel flat detection system is gradually moving towards device simplification, multi-sensor fusion, high algorithm accuracy, and operational intelligence. With continuous development of machine learning algorithms and constant perfection of railway databases, wheel flat detection based on machine learning algorithms will be the development trend in the future.

## 1. Introduction

**Figure 1:**
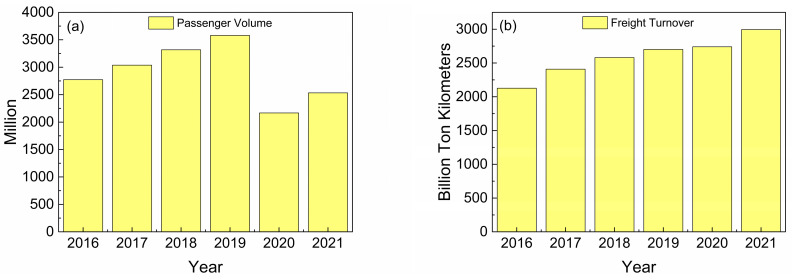
(**a**) Total railway passenger volume in China in 2021. (**b**) Total railway freight turnover in China in 2021.

Railways are considered to be one of the most important means of transportation at present. In China, the proportion of railway travel in people’s daily life has increased year by year. In 2021, China’s railway passenger volume has reached ~2.5 billion with an increase of 36.6 million over the previous year and a year-on-year increase of 16.9%, as shown in Figure 1a. In the same year, the total turnover of railway freight transportation reached ~2995.0 billion tons, with an increase of 255.2 billion tons over the previous year and a year-on-year increase of 9.3%, as shown in Figure 1b [1].

**Figure 2:**
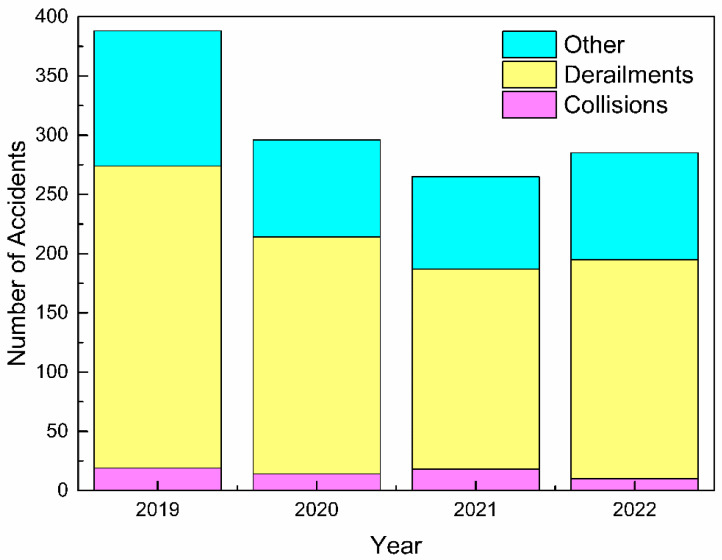
Main categories of railway failures in the United States from 2019 to 2022.

The demand for railway transportation has increased year by year, and the safety of railway transportation has become more and more important. According to the statistics of the United States Federal Railway Administration, 1234 train accidents were reported from January 2019 to February 2022 [2]. Among them, train derailments are the most common problems, with a total of 809 cases, accounting for 66% of the total number of failures, as shown in Figure 2.

As the most important running part of the train, the condition of railway wheels is closely related to the safety of train operation. Between 2005 and 2010, derailment accidents caused by rolling stock failures accounted for the highest proportion of accidents (38%) in 14 European countries (Austria, France, Germany, the UK, Sweden, Switzerland, Belgium, Bulgaria, Czech Republic, Hungary, Italy, the Netherlands, Poland, and Slovenia) [3]. Wheel flats are the most common local surface defect of railway wheels and are one of the important causes of train derailment [4]. Huge impact forces will be generated by the contact between flat wheels and rails, which will cause further damage to vehicles and rail components (wheel sets, bearings, rail ties, and so on) [5,6,7,8,9,10,11,12]. The excessive wheel–rail contact forces will increase the risk of train accidents and maintenance costs.

Wheel flats are caused by the loss of wheel tread material due to wheel slipping events [13]. Heat is generated as the wheels sliding on the rails, and the resulting temperature increase combined with the rapid cooling of the adjacent material can lead to the formation of brittle martensite on the wheel tread. Thermal impacts and phase transformations can generate large residual stresses, which will interact with the rolling contact stress to promote the formation and growth of cracks on the wheel tread [14]. There are many reasons for the wheels to slide on the rails, including (1) The sudden braking of the wheels when driving at high speed, thus causing the wheels to lock up and slide on the rail while the train is still moving [15]. (2) There is a local area where the wheel-rail friction becomes low. When there are foreign objects such as grease and leaves on the track, the wheel–rail adhesion is reduced, resulting in complete sliding between the wheel and rail [16,17,18,19]. In order to address the issue of wheel flats, the railway department has implemented various preventive measures, such as installing advanced anti-sliding systems on passenger trains. Despite these efforts, wheel flats cannot be completely eliminated. Therefore, wheel flat detection is considered to be an important measure of ensuring railway operation safety and reducing maintenance costs.

Human inspection has been the most common means of wheel flat detection in past decades. However, this method is time-consuming and prone to human errors. In-service testing methods can realize real-time detection of wheel flats without dismantling the wheelsets, and they have been studied by many researchers in recent years [20,21,22,23]. In-service testing methods can be divided into the on-board method and wayside method, according to different sensor installation positions. These two methods have their own advantages and disadvantages, and the selection of the method is based on a comprehensive consideration of the inspection duration, fault severity level, and so on [24,25,26]. In the on-board method, sensors are mounted on the axle box or wheels of a train, and different types of signals can be obtained by corresponding sensors such as accelerometers, microphones, etc., for analysis [27,28,29,30,31,32,33,34,35]. The flat signal obtained by the on-board method has strong periodicity and better robustness, which is conducive to signal processing and wheel flat detection. In the wayside method, sensors are usually installed on or nearby both sides of the track near train entrances or exit stations; the condition of all wheels can be evaluated as the train passes through the sensor system [36,37,38]. Unlike the periodic signals collected by the on-board method, each wheel can only be detected once by the wayside method, which leads to limited flat information and thus puts forward a high requirement for flat signal processing algorithms [39,40,41,42].

In 2017, Alemi and Corman reviewed the condition monitoring approaches for the detection of railway wheel defects [43]. In the recent few years, wheel flat detection has gained the attention of many researchers and flat detection techniques with new features were proposed, thus the summary and comparison of these techniques are of great significance. In this paper, the development of wheel flat detection techniques and corresponding signal processing methods with wayside deployment since 2016 are reviewed. The rest of this paper is organized as follows. The general structure of wayside in-service flat detection systems is demonstrated in Section 2. Wheel flat detection techniques proposed in the past five years is summarized in Section 3 and Section 4. In Section 5, the merits and weaknesses of the mentioned techniques are discussed and concluded.

## 2. Structure of Wayside In-Service Flat Detection System

**Figure 3:**
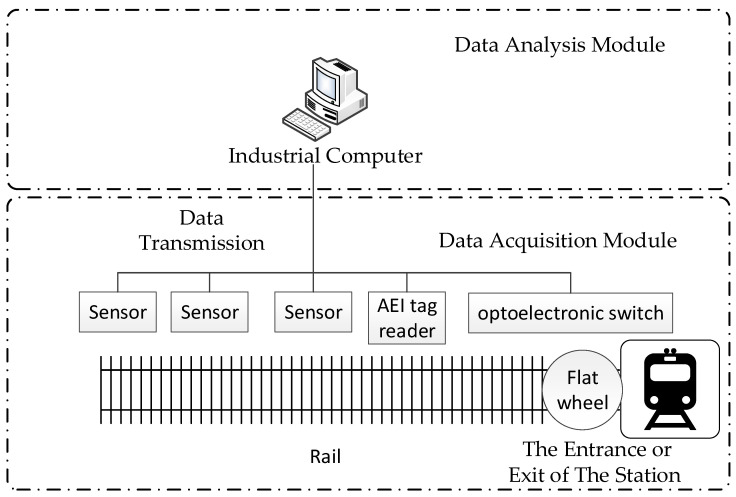
Wayside in-service flat detection system schematic.

Wayside detection systems are commonly used in automated rolling stock inspection processes [43,44,45,46]. The general structure of wayside in-service flat detection systems for railway vehicles is shown in Figure 3. It can be divided into two main parts: the data acquisition module and the data analysis module [47]. The data acquisition module is usually installed in the work room beside the rail to acquire wheel flat information when the train passes. Cameras, microphones, pressure sensors and other kinds of sensors can be used in data acquisition the module to sense flat information.

When the train passes the wayside detection system, the sensor installed beside the rail, such as an optoelectronic switch, will generate a ‘train coming signal’ to start the system. Automatic equipment identification (AEI) tag readers are often mounted on rails along with sensors to detect the wagon IDs and identify each wheel. The signal processor and the control PC then record the data collected from each axle in a database, which are then transmitted to the railway control center or depot maintenance center for remote monitoring and diagnosis [48,49]. The data analysis module is usually installed in the monitoring room beside the track. The signals generated by the data acquisition module are sent to the data analysis module via wired or wireless data transmission [50]. The signals are pre-processed to reduce noise interference and processed by wheel flat algorithms to extract flat information.

## 3. Stress-Based Wheel Flat Signal Acquisition Method

The most commonly used wheel flat detection method is the stress-based method. In this method, the dynamic stress of the track when the train passes can be measured by different stress sensors such as strain gauges, accelerometers, and fiber Bragg gratings (FBG) [51,52,53,54,55,56,57,58,59,60,61,62,63,64,65].

In 2016, Matthias Asplund et al. checked the wheel profile parameters measured by the wayside wheel profile measurement system (WPMS) installed along the Swedish Iron Ore Line, and correlated them with the warning and alarm indications issued by the wheel defect detector (WDD) [66]. The WDD measures the force peak generated by static train load and dynamic load from the wheel defects [67]. WPMS can detect faults related to wheel profile, such as high flange, wide flange, thin flange, small flange angle, and abnormal wheel diameter. WDD can detect faults such as flats, large shelling, and severe wheel polygonization. Unfortunately, none of these systems can successfully capture surface cracks, spalling, small shelling, low levels of polygonization, or all subsurface defects, nor can they detect rolling contact fatigue (RCF) in wheels at an early stage [68,69].

**Figure 4:**
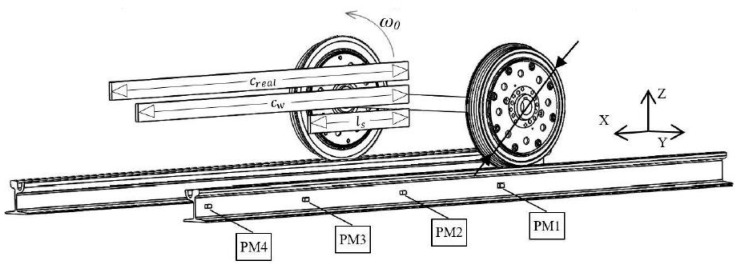
A view of the measurement points locations with a basic designation of dimensions and measurement realization. ω0: rotational velocity of the wheel, PM1~PM4: points of measurements [70].

In 2018, several vibration transducers were mounted on tracks by Tomasz Nowakowski et al., to detect the wheel flat as shown in Figure 4 [70]. A vibration signal processing method based on time-domain and frequency-domain was proposed. By analyzing the time signal envelopes of flat-wheel and ordinary-wheel trams, obvious peaks were found in the time signal envelopes of flat-wheel trams. They measured 15 tram passes with flawless wheels and 17 tram passes with flat wheels and found the time signal envelope of the flat wheel tram is characterized by an obvious peak with an amplitude higher than 35 m/s^2^, while the time signal envelope of the flawless tram does not have this feature. Experimental results show that this method has high efficiency in wheel flat detection, and can be applied to a larger speed range.

Liu and Ni developed an FBG-based track-side wheel condition monitoring system for detecting wheel tread defects [71]. Two FBG strain gauge arrays mounted on the foot of the track were used to measure the dynamic strain of paired tracks excited by passing wheelsets. Each FBG array was approximately 3 m in length, slightly longer than the wheel circumference to ensure full coverage to detect any potential defects on the wheel tread. A defect detection algorithm was developed that utilizes online monitored rail responses to identify potential wheel tread defects. Data smoothing techniques were used to detrend and to pre-process the strain data and outlier analysis was used to perform diagnostics of responses to normalized data. Local defects can be identified by refined analysis of responses extracted in diagnostics. According to field tests, the proposed method can achieve satisfactory accuracy in wheel defect detection when the train running speed was higher than 30 kph, and some minor defects with a depth of 0.05 mm~0.06 mm were also successfully detected.

In the same year, Gabriel Krummenacher et al. proposed a wheel flat detection system based on the measurement of vertical force through wheel load checkpoints (WLC) installed on the rail [72]. Each WLC consists of four 1 m long measurement bars with four strain gauges per measurement bar. The strain gauges were installed perpendicular on the centerline of the rails. An automatic detection and classification method for railway wheel defects based on vertical force sensors was proposed. Wavelet features of time series data from sensors were designed and support vector machines were used as classifiers. Convolutional neural networks (CNN) for different wheel defect types were designed and trained through deep learning. A cyclic shift invariant artificial neural network was designed to detect flat and non-round wheels. The proposed method can be used to predict wheel defects without prior knowledge of how these defects will manifest in measurements.

**Figure 5:**
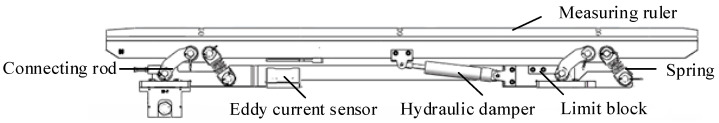
A schematic diagram of the parallelogram mechanism [73].

In 2019, a railway wheel flat detection system based on a parallelogram mechanism was improved on the basis of the previous research of our group [73]. As the core component, the parallelogram mechanism is mainly composed of the measuring ruler, connecting rods, springs, hydraulic damper, limit block, and eddy current sensor, as shown in Figure 5. The wheel flat can be quantitatively detected by measuring the vertical displacement change of the measuring ruler. The depth of the wheel flat can be reflected by the amplitude of the sensor signal.

Wen-Jun Cao et al. used the dynamic signal of rail pad sensors (RPS) to identify the wheel flats [74]. A method to identify wheel flat dimensions using a dynamic measurement-based model update strategy was proposed. They used a model falsification approach to identify the size of the wheel flats, which can interpret high-dimensional time series in the context of inverse identification. This method has been successfully applied to process time series data and significantly reduces computation time. The system was field-tested on a test track at a train station in Singapore and the experimental results showed that the identified wheel flat size is within the real observation range.

In the same year, Alemi A. et al. proposed a fusion method for wheel defect recognition, which associates the collected samples with their positions on the wheel circumference coordinates [75]. As the magnitude of the contact force contains limited information about wheel defects, this study reconstructs defect signals from discrete samples collected by multiple sensors, such as WILDs. The obtained results show a considerable similarity between the contact force and the reconstructed defect signal, which can be used for further defect identification. The proposed method offers the potential to detect and identify defects at an early stage, including minor defects and long-wave defects. In addition to wheel defects, the reconstructed defect signal will also be influenced by other parameters, such as train velocity, axle load, number of sensors, and wheel diameter. In 2020, they carried out a parameter study to investigate the impact of these parameters [76]. The research shows that the fusion method can provide better performance when the signal-to-noise ratio (SNR) is high. Increasing the number of sensors can improve the results of the fusion process. Therefore, it is necessary to balance the cost of the interrogator supporting a large number of sensors with the accuracy and reliability of the fusion results.

**Figure 6:**
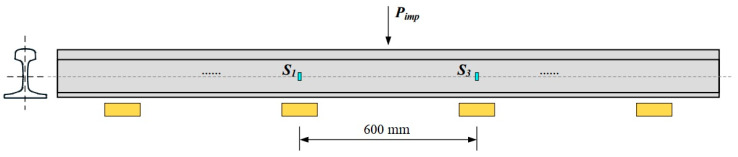
Plans for sensor arrangement [77].

In 2020, Chenyi Zhou et al. proposed a long-term monitoring method for wheel flats based on multi-sensor arrays, as shown in Figure 6 [77]. The dynamic strain response of the rail was captured efficiently by an array of sensors mounted on the rail web to ensure that all wheels were evaluated during the passage of the train. In order to realize accurate recognition and positioning of wheel flats, an algorithm based on multi-source data fusion was proposed. A vehicle–track system coupling dynamic model was established, and the sensitivity and reliability of different sensor layout schemes under different wheel flat conditions can be analyzed according to the model. By conjoint analysis of multi-sensor signals, the specific moment at which the wheel flat occurred can be precisely identified. By use of data fusion between multiple sensors, the specific location of wheel defects can be confirmed.

**Figure 7:**
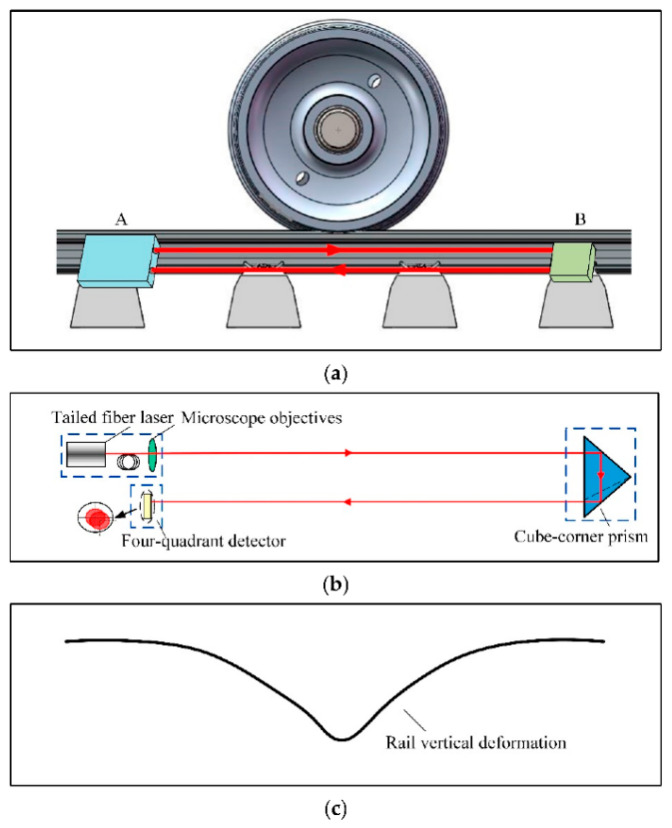
The schematic diagram of the sensor and the rail vertical deformation: (**a**) The installation location of the sensor; (**b**) The schematic diagram of the sensor; (**c**) The rail vertical deformation [78].

Our group designed a new wheel flat detection system based on a self-developed reflective optical position sensor [78]. As shown in Figure 7, the sensor was composed of a tailed fiber laser, a four-quadrant detector, and a cube-corner prism. A in Figure 7a represents the light source and detection module composed of a tailed fiber laser and a four-quadrant detector. B in Figure 7a is a reflection module composed of cube-corner prism. The reflective optical position sensor is used to detect the vertical deformation of the rail under wheel–rail contact. The wheel–rail impact force of the entire circumference was measured by displacement detection of the collimated laser spot. The finite element method and multibody dynamics method were used to establish the vehicle–track coupled dynamic analysis model. A quantitative relationship between the sensor signal and the wheel flat length was established by the model. The system was assessed through simulation and laboratory investigation, and real field tests were conducted to certify its validity and correctness.

A vibration signal-based flat detection system was developed by Jyoti Barman and Durlav Hazarika [79]. In this system, the vibration signal was captured by an ADXL335 vibration sensor connected to the fish-plate of the track and linear time-frequency transform (wavelet transform) combined with a quadratic time-frequency transform (Wigner-Ville transform) were used to find the flat signal.

**Figure 8:**
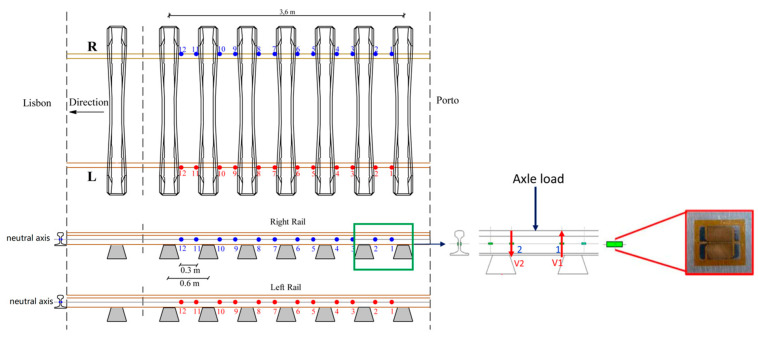
Strain gauges’ positions [80].

In 2021, Araliya Mosleh et al. proposed a wheel flat detection system based on strain gauges (SGS) mounted on the trackside which has been tested in several scenarios of varying complexity [80]. The layout scheme of the strain gauges is shown in Figure 8. The numbers in the figure represent the deployment position of the strain gauge on the rail. A method using envelope spectrum analysis to detect wheel flats was proposed. Through envelope spectrum analysis, wheel flats at different train speeds can be detected if a noticeable lag between the amplitudes of the envelope spectrum is observed. In 2021, they proposed a multi-sensory layout scheme to detect wheel flats on passenger and freight trains [81]. The effect of sensor type and installation location on the accuracy of the wheel flat detection system was analyzed and discussed. Experimental results show that using a layout scheme consisting of accelerometers is clearly more beneficial than using strain gauges to perform envelope spectrum analysis to detect defective wheels in situations where the signal is heavily contaminated by noise. Compared with the previous study, the number of sensors was reduced and the installation positions of sensors were optimized.

Ni and Zhang established an FBG-based wayside monitoring system by deploying two arrays of FBG strain sensors beside the track [82]. Each sensor array includes 21 FBG gauges, and each gauge is evenly spaced at 0.15 m intervals on the rail foot of each single track. A Bayesian machine learning approach based on trackside strain-monitoring data was developed for online and quantitative assessment of railway wheel conditions. The cumulative density functions of the normalized Fourier amplitude spectra of the rail foot strain response under healthy wheel conditions were extracted as features. The probabilistic reference model was trained by Sparse Bayesian Learning (SBL). Due to the sparsity of SBL embedding, overfitting was avoided and the generalization ability was improved. As only a small number of basic functions were involved in the model, the computational efficiency of the model was competitive, and fast diagnosis can be realized in wheel condition assessment. The proposed method is verified by using the in-situ monitoring data collected by the wayside monitoring system during the train passing process. The proposed method is verified by comparing the diagnosis results obtained from the proposed online method and the offline wheel radius deviation measurement.

In 2022, Jian Mu et al. studied the dynamic behavior of the vehicle system and the contact force between the wheel and the rail [83]. The detection of wheel–rail vertical contact force was realized by the prototype through rail web strain gauges. Then a vehicle–rail coupling model considering the modal characteristics of the flexible wheelset and the track was established. The validity of the fitted curve to determine the flat length was checked by comparing the simulated plane length with the length calculated for the wheel–rail contact force.

Araliya Mosleh et al. proposed a data-driven machine learning method based on unsupervised learning, which can automatically differentiate between defective and healthy wheels of a train [84]. This method combines sets of acceleration and shear force records evaluated on the rail to enhance its sensitivity. By taking one or more train passing signals with different operating speeds, loading schemes, and track irregularities profiles as input, an artificial intelligence method was used to identify wheel flats. A continuous wavelet transform model was employed to extract features from multiple sensors, transforming time series measurements into alternative data. Then, the extracted features were normalized using Principal Component Analysis techniques, suppressing environmental and operational variations. Finally, data fusion was employed to merge the features from each sensor and enhance the sensitivity to detect wheel defects.

## 4. Sound- or Image-Based Wheel Flat Signal Acquisition Methods

In addition to stress-based methods, many sound- and image-based methods have also been used to detect wheel flats [85,86,87,88].

### 4.1. Sound-Based Method

**Figure 9:**
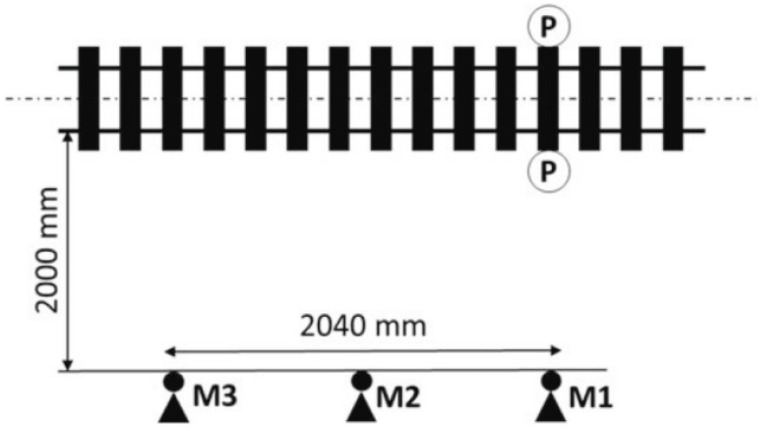
The scheme of measuring positions in the pass-by test; M—Microphones, P—photocells [89].

**Figure 10:**
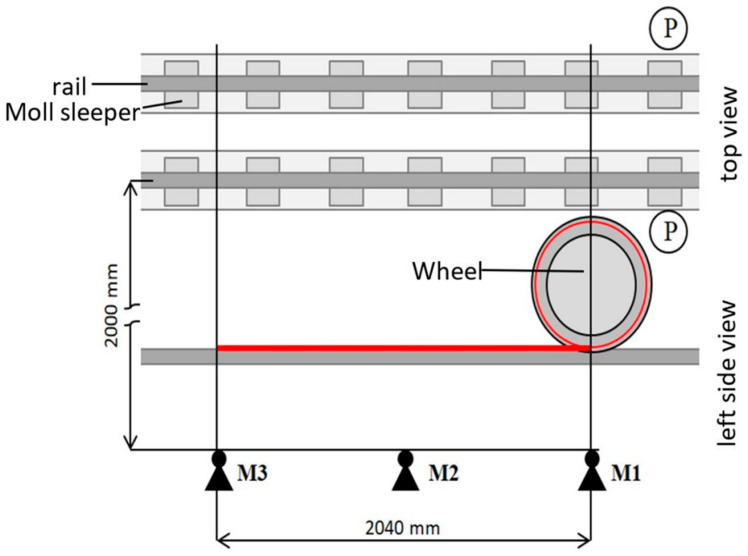
The scheme of measuring positions during acoustic pass-by tests; M1–M3—Microphones, P—Photocells [90].

When the wheel flat appears on the wheel tread, the wheel–rail contact force will become uneven and impact rolling noise will be generated. In the sound-based method, acoustic sensors such as microphones and acoustic emission (AE) sensors are installed on the side of the track to acquire flat signals. In 2017, Pawel Komorski et al. realized the identification of wheel flats by detecting collision noise generated by wheel–rail contact [89]. The layout scheme of the measurement system is shown in Figure 9. Three microphones were installed on one side of the track and spread along the track with a distance of 2.04 m, which is the length of the circumference of each tram wheel. A transmitter-receiver type photocell was placed between the tracks to measure the cross-section. The joint time-frequency analysis method (JTFA) was used to process acoustic signals and detect the flat wheels of trams, which consists of short-time Fourier transform (STFT) analysis and wavelet transforms. Subsequent studies have shown that the total cost can be reduced by reducing measurement points [90]. The improved system layout scheme is shown in Figure 10. The acoustic signal was analyzed according to the Fourier transform and the Hilbert transform. The novelty of this method is the use of acoustic signals instead of vibration signals to estimate the diagnostic parameters of the second and third rotational harmonic frequencies of the wheel. The highest sensitivity was obtained at the level of 13–15 dB.

In 2018, Metin Aktas et al. detected wheel flats based on acoustic emission (AE) technology and the parametric constraint optimization principle [91]. In the system, a single AE sensor was used and deployed on rails equipped with magnetic holders. As the train moves on the rail, the produced acoustic vibration can be measured continuously. A wheel flat detection algorithm based on parametric constraint optimization principles was proposed. This method compares the measured defect score curve with the predefined threshold curve to determine the wheel tread condition. Field tests were conducted and the results show that the system can effectively detect wheel flats at different train speeds with an accuracy rate of up to 90%. The challenge of this method is that the measured acoustic signal may contain the surrounding noise, which limits the method’s accuracy.

### 4.2. Image-Based Method

With the development of computer vision technology, many image-based wheel flat detection systems have been designed [92,93]. In these systems, high-speed cameras were used to acquire a photo of the wheel tread when the train passes by. Then the wheel tread defects can be identified and localized by corresponding image processing algorithms.

In 2016, Hanieh Deilamsalehy et al. developed a computer-vision-based system for automatically detecting the sliding wheels and hot bearings from images taken by wayside thermal cameras [94]. From the acquired thermal images and sliding wheels, it can be seen that the sliding wheels possess a distinctive heat pattern at the wheel–track contact point. A method based on histograms of oriented gradients for identifying wheel flats and bearing parts was proposed. The feature descriptors were used by support vector machines to build fast classifiers with good detection rates. Simulated images of sliding wheels were used to train the algorithm. The monitoring method was tested with simulated images and a set of real thermal images taken on several trains of the Union Pacific Railroad (UPPR). 98% of the total number of defective wheels were detected without any false alarm. The model was improved by the author in the follow-up work, and the accuracy reached 100% [95].

In 2017, a system of collecting wheel tread defect images for online running trains was designed by Guangyu Guo et al. [96]. 16 high speed CCD cameras were used in the system to acquire the images of the entire wheel tread. By Support Vector Machine (SVM) classifier and Gaussian kernel, the wheel tread defect areas can be identified and located accurately. A wheel tread flat detection method was proposed to deal with wheel tread images captured by high-speed cameras. In this method, an SVM classifier was employed to recognize defective areas and a Gaussian kernel was used to locate defect areas. Three different types of descriptors were extracted to represent the defects, and experimental results show that compared with distribution vectors and area features, HOG features were considered to be better features for defect identification.

The image-based method uses vision cameras as flat sensors, which has the characteristic of non-contact measurement and fast response time. However, the accuracy of this method depends on image processing algorithms.

## 5. Summary

The advantages and disadvantages of the three signal acquisition methods are shown in Table 1. Currently, the sound-based method has been applied widely in non-destructive tests, but it is not a popular solution for wayside wheel flat detection [90]. The stress-based method is the most commonly used wheel flat detection method as its advantages of low cost, convenient installation, and high accuracy [89]. However, the traditional wheel flat detection system based on the stress method can only detect the wheel–rail contact area, which will cause more than 70% of the wheel tread area to be undetectable [96]. In addition, the size of the flat cannot be directly reflected by the acquired waveform, thus it is necessary to build models to acquire the relationship between flat size and the waveform. As models do not necessarily reflect the real field conditions, the quantitative measurement of wheel flats remains a challenge for the stress-based method. Generally, the image-based method is more expensive due to the use of lasers or cameras. In this method, the wheel flat size can be identified directly and quantitatively through image processing algorithms.

## 6. Conclusions

**Figure 11:**
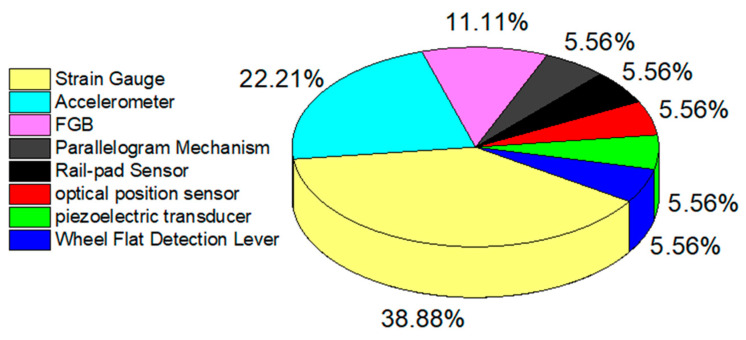
Usage statistics for different sensors.

This paper discusses the research progress in wayside wheel flat detection since 2016 and compares the advantages and disadvantages of various wheel flat detection methods. During this period, the stress-based method is the most popular flat detection method. Strain gauges, accelerometers, and FBG are commonly used stress sensors to extract rail vibration signals. According to the Literature referenced in this paper, the application statistics of sensor types in stress-based wayside wheel flat detection systems in the past few years are shown in Figure 11. Different sensors have different characteristics, which can be flexibly selected according to the requirements. The strain gauge has the advantages of low price and high resolution, however it is non-linear and needs regular calibration. In addition, the measuring results are easily affected by external factors such as train speed, train weight, and electromagnetic interference. Therefore, more simulation studies are needed to evaluate the impact of train speed, train weight, and severity reflected in wayside measurement data. The FBG sensor has high precision and is immune to EMI induced noise. With wavelength multiplexing capability, multiple FBG sensors can be integrated, which is convenient for remote monitoring, deployment, and maintenance. The main challenges faced by FBG sensor applied in wheel flat detection include: ① Certain cross-sensitivity in strain and temperature response exists in FBG sensors; ② Currently, most of the systems based on FBG sensing have a measurement speed below 1 kHz. However, for high-speed measurement scenes such as wheel flat detection, the signal demodulation speed of FBG sensing-based schemes needs to be further improved. In addition, the study of new structure and packaging of FBG sensors has important significance. To address the issue of cross-sensitivity, researchers have put forth several temperature compensation models and algorithmic solutions [97]. In addition, it is also of great significance to study new structural designs and packaging options of FBG sensors to solve this problem. At present, the main factors restricting the detection speed of FBG signals are the modulation rate of the light source and modulator, and the response frequency of the detector. By improving the speed level of these devices, high-speed detection ability will be further improved. For FBG signal demodulation methods, spatial dispersion spectroscopy and time dispersion spectroscopy with higher sampling rates will be good choices. In addition, adopting the approach of collecting data first and processing them later can reduce the requirements for the signal analysis and processing capabilities. The research of effective installation methods and cost-effective high-performance demodulation systems for FBG sensors promote the application of FBG in the detection of wayside wheel flats. In addition to the above usual stress sensors, laser measurement technology has been introduced as a stress sensor which has brought many new ideas for wheel flat detection.

Machine learning has been attracting more and more attention in the field of wheel flat detection. Machine learning algorithms, such as deep learning, can learn patterns from wheel flat signals, enabling them to realize self-feature extraction which can better capture flat signals. In addition, they are able to adapt to changing fault conditions, allowing them to perform more robustly when faced with new fault situations. Compared with the traditional threshold method, machine learning algorithms have higher accuracy but slightly lower precision [72]. This means that the threshold method has a low false alarm rate but is prone to miss detections, resulting in partially flat wheels being ignored after passing through the detection system. Compared with the traditional threshold method, machine learning algorithms have a high recall rate, indicating that they are less likely to miss detections of wheel flats. However, the application of machine learning also faces challenges. The performance of machine learning algorithms is mainly influenced by the model structure, the distribution of training data, and hyper parameters. A complex model structure and a large amount of training data greatly increase the cost of model training. In the real world, the amount of data for faulty wheels is severely imbalanced compared to that of normal wheels, with labeled data shortages decreasing the model’s performance and robustness. Few-shot learning and unsupervised learning models have great potential in addressing these issues. Additionally, in order to ensure the generalization ability of machine learning algorithms and to make them applicable to in-service wheel flat detection on different railways, it is necessary to construct larger and more reasonable data sets. Appropriate model structures and well-distributed data sets can not only guarantee detection accuracy, but also ensure detection robustness by reducing overfitting. This may promote machine learning algorithms as the main development direction for wayside wheel flat detection.

In addition, by combining stress-based sensors, which are sensitive but have limited measurement range, and image-based sensors, which have a large measurement range but are easily affected by attached objects such as leaves and soil, multi-sensor fusion measurement can be achieved, which can further improve the sensitivity and accuracy of the measurement. Multi-sensor fusion can locate the specific position of wheel defects, but its results are affected by parameters such as the number of sensors and the length of the effective area [75,76,77]. By analyzing these parameters, multi-sensor fusion may have greater development potential in the field of wheel flat detection. The development direction of the wheel flat detection system may gradually tend to device simplification, multi-sensor fusion, algorithm accuracy and operation intelligence. In addition to single fault detection, the detection system is also gradually adopting multi-fault detection to be more efficiently. Apart from the wheel flat detection system, there are also wheel tread geometric parameter detection systems, wheel diameter detection systems, and so on [98,99]. Multi-system combined wheel status detection will become the future development trend.

## Figures and Tables

**Table 1:** Advantages and disadvantages of stress-based, sound-based, and image-based techniques.

Method	Advantages	Disadvantages
Stress-based method	Related technologies are more mature	It can only detect the condition of the wheel–rail contact area
	Simple installation and maintenance	
	Low cost	Quantitative measurement is difficult
Sound-based method	Acoustic emission method can realize repeated period measurement	Quantitative measurement is difficult
	Low cost Easy to use	Relative technology application is less
Image-based method	Quantitatively measurable, Non-contact measurement, Long life	High cost

## Data Availability

No new data were created or analyzed in this study. Data sharing is not applicable to this article.
